# Mechanistic Study on the Optimization of Asphalt-Based Material Properties by Physicochemical Interaction and Synergistic Modification of Molecular Structure

**DOI:** 10.3390/polym16202924

**Published:** 2024-10-18

**Authors:** Jiashuo Cao, Lifeng Wang

**Affiliations:** School of Civil Engineering and Transportation, Northeast Forestry University, Harbin 150040, China

**Keywords:** fiber-modified asphalt, fiber chemical structure, rheological properties, molecular dynamics

## Abstract

In order to investigate the relationship between the molecular structure of fibers and the differences in physicochemical interactions between fibers and asphalt on the performance of fiber-modified asphalt, this paper chose two types of fibers with different chemical structures: straw fiber and polyester fiber. First, the differences in molecular interactions between the two fibers and asphalt were explored using molecular dynamics, then the differences in the adsorption capacity of the two fibers on asphalt components were tested by attenuated total reflection infrared spectroscopy experiments, and finally, the differences in the rheological properties of the two fiber-modified asphalts were tested by dynamic shear rheology and low-temperature creep experiments. The molecular dynamics simulation findings reveal that polyester fibers may intersperse into asphalt molecules and interact with them via structures such as aromatic rings, whereas straw fibers are merely adsorbed on the asphalt’s surface. Straw fibers and asphalt exhibit hydrogen bonding, whereas polyester fibers and asphalt display van der Waals interactions. The results of attenuated total reflectance infrared spectroscopy indicated that polyester fiber absorbed asphalt components better than straw fiber. The rheological tests revealed that the polyester fiber had the highest complex shear modulus in the temperature range of 46–82 °C, and at 64 °C, the phase angle was 4.289° lower than that of the straw fiber-treated bitumen. Polyester fiber-modified asphalt had a 32.48%, 15.72%, and 6.09% lower creep modulus than straw fiber-modified asphalt at three low-temperature conditions: −6 °C, −12 °C, and −18 °C. It is clear that fibers with aromatic rings as a chemical structure outperform lignin-based fibers in terms of improving asphalt characteristics. The research findings can serve as a theoretical foundation for the selection of fibers to produce fiber-modified asphalt.

## 1. Introduction

Nowadays, asphalt-based materials are widely used due to their excellent adhesion and waterproofing properties, such as asphalt waterproofing materials in the field of construction [1] and asphalt binders in the field of road engineering, which play the role of adhesion [2,3,4]. However, as a viscoelastic material, the viscoelastic properties of base asphalt are very dependent on temperature changes [5,6], and an increase in temperature will gradually reduce the viscosity of matrix asphalt. In addition, the base asphalt itself has limited viscosity. Therefore, researchers have gradually modified the asphalt by adding modifiers to improve the viscosity and temperature sensitivity of the matrix asphalt.

In terms of modifier selection, researchers have added modifiers (e.g., polyphosphoric acid, nanomaterials, etc.) to asphalt in order to improve the properties of asphalt [7,8,9]. Xiao F et al. [10] explored the possibility of polyphosphoric acid for use as a modifier in place of styrene–butadiene–styrene and tested the properties of modified asphalt. The test results show that the nature of the polymer itself affects the properties of the modified asphalt, with the base binder having a greater influence. In addition, nanomaterials can adsorb more asphalt due to their large specific surface area, thereby improving the properties of asphalt. Moghadas Nejad F et al. [11] added different dosages of calcium carbonate nanoparticles to asphalt mixtures and tested the properties of asphalt mixtures before and after modification. The test results found that the incorporation of calcium carbonate nanoparticles can improve the high-temperature performance of asphalt mixtures, extend the fatigue life, as well as increase the resistance of asphalt mixtures to water damage. Zeng Q et al. [12] modified asphalt with graphene oxide as a modifier and tested the performance changes and chemical composition changes of the modified asphalt. The results of the study show that the high-temperature performance and thermal aging resistance of asphalt can be significantly improved by incorporating graphene oxide into asphalt, and graphene oxide can form an interpolated structure in asphalt. However, the test results of infrared spectroscopy showed that the mechanism of graphene oxide-modified asphalt was only physical adsorption and no chemical reaction occurred.

In addition to polymers and nanomaterials that can be used as modifiers, in recent years, fibers have gradually been taken into account by researchers due to their ability to adsorb more asphalt as well as the reinforcing effect of fibers on asphalt [13,14], and the performance of fiber-modified asphalt has been gradually explored. Arabani M et al. [15] tested the macroscopic properties such as viscosity and crack resistance of ceramic fiber-modified asphalt. The test results showed that the incorporation of ceramic fibers can effectively improve the cracking resistance of asphalt at low temperatures as well as the adhesion under high-temperature conditions. Additionally, the optimum dosage of 3% was determined. In addition, the study of Al-Kheetan M J et al. [16] confirmed that ceramic fiber-modified asphalt constitutes physical modification, i.e., it only relies on the adsorption of asphalt on the surface of the fibers and the formation of a three-dimensional mesh structure of the fibers in the asphalt to achieve the improvement of asphalt properties, and the modification process does not involve chemical action. Chen H et al. [17] made a comparison between the performance of polyester fiber-modified asphalt and plant fiber-modified asphalt such as lignin and asbestos fibers and tested the differences in the fiber mesh structure within the asphalt and the effect on the macroscopic properties by scanning electron microscopy. The test results show that polyester fibers can form a larger three-dimensional mesh structure within the asphalt due to their own fiber structure, but plant fibers such as lignin and asbestos fibers can adsorb more asphalt due to their larger specific surface area. In addition, both polymer fibers and plant fibers can improve the high-temperature properties such as the rutting resistance of asphalt. However, this comparison only stays in the aspect of macro performance, and no research is undertaken from the aspect of the modification mechanism. Li Z et al. [18] explored the differences in the performance of a variety of straw fiber-modified asphalts, which observed through scanning electron microscope experiments that the different internal structure of the fibers affects the content of the structural asphalt adsorbed by them; in addition, the incorporation of plant fibers can likewise improve the toughness of the asphalt and the resistance to rheological properties. In addition, Guo R et al. [19] explored the variability of the rearrangement of different components of asphalt on the fiber surface from the perspective of adsorption kinetics, and the results of the study showed that the fibers can adsorb components of asphalt by physical cross-linking or chemical cross-linking effects.

Existing research results have reached a consensus on the conclusion that fiber surfaces can adsorb asphalt, but due to the different mechanisms of adsorption, such as physical adsorption by van der Waals forces only and physicochemical synergistic adsorption, there must be differences in the properties of the modified asphalt. For physical adsorption, molecular dynamics can be evident in physical adsorption such as in intermolecular hydrogen bonding and van der Waals interactions [20,21]. Cui W et al. [22] used molecular dynamics to investigate the adsorption of asphalt components on the surface of carbon nanotubes, and the results showed that the type of asphalt components adsorbed on carbon nanotubes depended on the size of the molecular weights of the asphalt components, and the ones with small molecular weights were more likely to be adsorbed on the surface of carbon nanotubes.

Based on the above-mentioned physical–chemical mechanism of adsorption of asphalt on fiber surfaces, the differences in the effects on the properties of modified asphalt are still unclear. In this paper, two kinds of fibers, polyester fiber with active groups on the surface and straw fiber without active groups on the surface, were selected to prepare fiber-modified asphalt, and the differences in the distribution of asphalt components on the surface of the two kinds of fibers under the effect of physical adsorption were analyzed by molecular dynamics. At the same time, this is combined with the second-order derivative infrared spectroscopy technology to analyze the differences in chemical adsorption and finally combined with the differences in the macroscopic properties of modified asphalt comparison to obtain the physical–chemical synergistic effect of modification and the single physical modification effect differences.

## 2. Materials and Methods

### 2.1. Simulation Methods for Intermolecular Physical Interactions

#### 2.1.1. The Establishment of the Molecular Modeling

**Figure 1:**
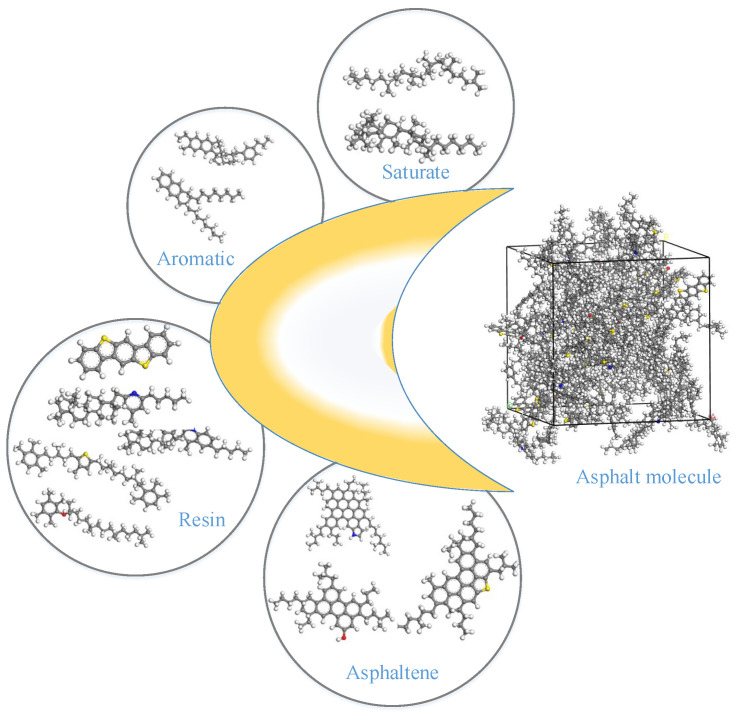
Asphalt components and molecular modeling of asphalt.

In this paper, we use the Materials Studio 8.0 software to construct asphalt molecules based on the AAA-1 asphalt model proposed by Li et al. [18]. The AAA-1 model contains four components, asphaltene, resin, saturate, and aromatic, and the molecular model of each component in the asphalt and the final constructed molecular model of asphalt are shown in Figure 1.

**Figure 2:**
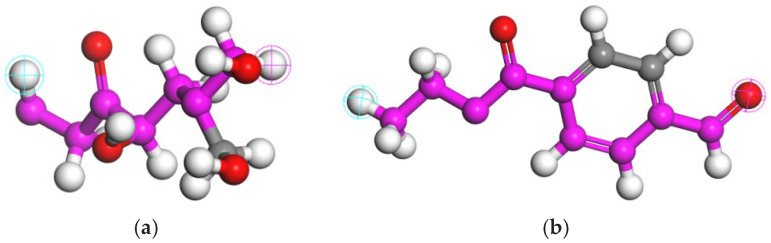
Molecular model of fiber monomers: (**a**) monomer modeling of straw fibers, (**b**) monomer modeling of polyester fibers. Note: Pink and gray are carbon atoms, red shows oxygen atoms, and white shows hydrogen atoms.

As noted by [23], polyester fibers are synthesized via polycondensation of dibasic acids and diols, while [24] highlights that straw fibers primarily consist of hemicellulosic substances, so the monomers of polyester fibers and straw fibers constructed in this paper are shown in Figure 2.

**Figure 3:**
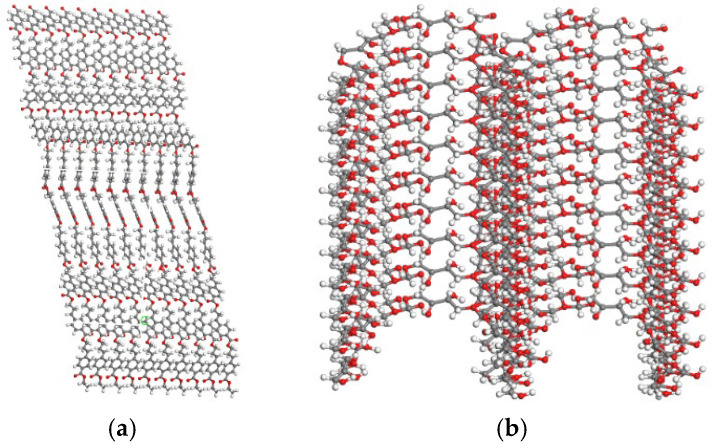
Structural model of the fiber molecule: (**a**) molecular modeling of polyester fibers, (**b**) molecular modeling of straw fibers.

In constructing polyester fiber and straw fiber molecules, in this paper, in order to simplify the calculation and save the cost of calculation, the degree of polymerization of both fibers was 5, while each fiber was repeated to construct 5 fiber chain-like structures. Figure 3 shows the final molecular model of the fiber.

#### 2.1.2. Validation of the Validity of Molecular Models

**Figure 4:**
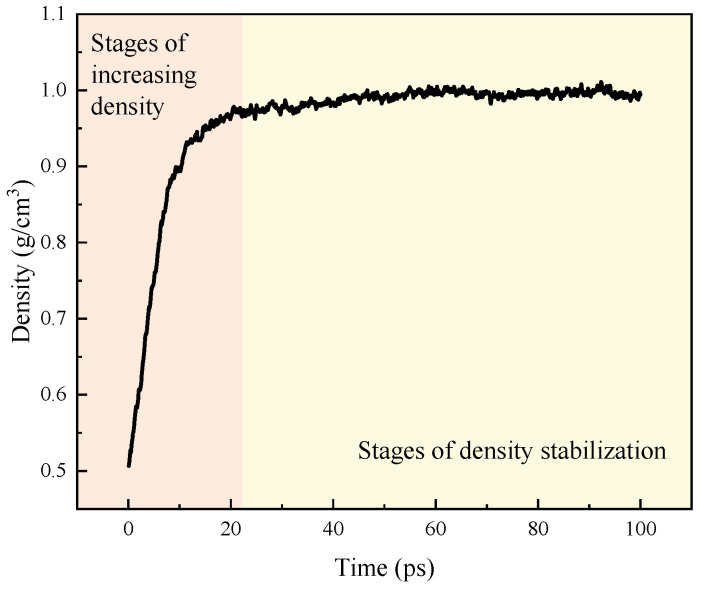
The process of density change of asphalt molecules.

In order to verify the validity of the constructed asphalt molecular model, in this paper, the initial density of asphalt molecules was set to be 0.5 g/cm^3^ and run in the NPT system at 298.15 K for 100 ps. The density variation during operation is shown in Figure 4.

The density change process in Figure 4 demonstrates that the density of the asphalt molecule model steadily increases from the initial 0.5 g/cm^3^ and ultimately stabilizes at 1.0 g/cm^3^. Upon comparing the density of the asphalt molecules constructed within this paper with the experimentally determined density of asphalt, it is evident that they are in good agreement with the density range of actual asphalt materials (spanning from 0.9 g/cm^3^ to 1.1 g/cm^3^). Consequently, the asphalt molecular model developed in this study holds the potential for simulating real-world asphalt systems.

#### 2.1.3. The Process of Molecular Dynamics Operations

The purpose of this paper is to analyze the interaction between two fiber molecules and asphalt molecules and to prepare fiber-modified asphalt in practice at a shear temperature of 180 °C; therefore, in this paper, the two constructed structural models of fiber asphalt were first geometrically optimized for 20,000 ps in order to make the energy of the fiber asphalt molecular model system minimized, and then they were run for 100 ps at a temperature of 453.15 K (180 °C) and in an NPT system in order to achieve the full contact between the fiber molecules and the asphalt molecules.

#### 2.1.4. Radial Distribution Function

The radial distribution function refers to the probability of the occurrence of another atom or molecule within a certain distance from the central atom, resulting in different distances between different atoms and the central atom due to the different strengths of hydrogen bonding and van der Waals interactions between the two atoms or molecules [25]. Thus, the radial distribution function describes the type of interaction between the core atom and the other atoms that surround it. The radial distribution function is calculated as shown in (1):

(1) g(r)=dNρ4πr2dr

where: r is the distance between particles, ρ is the density of the system, and N is the total number of particles.

#### 2.1.5. Mean Square Displacement and Diffusion Coefficient

In molecular dynamics, the thermal mobility of a molecule may be described by the mean square displacement of the molecule relative to the diffusion coefficient. The formula for calculating the mean square displacement (MSD) is shown in (2):

(2) $$x=\left\langle {\left|r_{i}\left(t\right)-r_{i}\left(0\right)\right|}^{2}\right.$$

The diffusion coefficient quantitatively describes molecules’ capacity to travel in Brownian motion. In molecular dynamics, the diffusion coefficient is computed using the slope of the mean square displacement curve [26]. The formula is shown in (3):

(3) $$D=\frac{1}{6}\underset{t\to \infty }{\lim}\frac{dx}{dt}$$

where: x is the value of the mean square displacement, $r_{i}\left(t\right)$ is the Particle displacement at time t (m), and $r_{i}\left(0\right)$ is the displacement of the particle at the initial moment (m).

#### 2.1.6. Relative Concentration Distribution

Relative concentration can characterize the spatial distribution of molecules [27]. As a result, in order to determine the distribution of the two types of fibers in asphalt, this paper calculated the relative concentration distribution of straw fibers and polyester fibers along the direction of asphalt (*z*-axis direction) using molecular dynamics in order to determine the difference in the size of the two types of fiber molecules in terms of their ability to penetrate into the asphalt molecules.

### 2.2. Material and Specimen Preparation

#### 2.2.1. Asphalt

The asphalt utilized in this research was 90# base asphalt made in Panjin, China (Panjin North Asphalt Co., Ltd.). It has been tested, and its technical parameters are provided in Table 1.

The statistics in Table 1 demonstrate that all indexes of 90# matrix asphalt utilized in this work meet the technical standards of the Technical Specification for Highway Asphalt Pavement Construction (JTGF40-2004) [28].

#### 2.2.2. Fibers

The two fibers used in this paper are polyester fiber and straw fiber, both of which are from Jiangsu, China. After testing, the technical parameters of the two fibers are displayed in Table 2 and Table 3.

**Figure 5:**
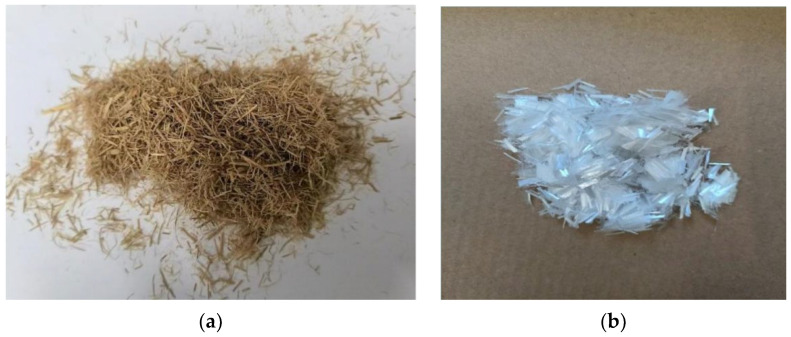
Two fiber material morphologies: (**a**) straw fiber, (**b**) polyester fiber.

The macroscopic morphology of the two fibers is shown in Figure 5:

#### 2.2.3. Preparation of Specimens

This paper is concerned with the influence of changes in the chemical structure and physicochemical interaction between the surface composition of the two fibers and the asphalt on the qualities of the asphalt. As a result, in the specimen preparation, the optimal dose of both fibers was chosen to make fiber-modified asphalt, as recommended by Chen K et al. [29]. The dose of both fibers was set at 3%.

In this research, two types of fiber-modified asphalt were made by first heating the asphalt to the flow state at 165 °C, and then utilizing a high-speed shear to shear the asphalt at a shear rate of 5000 r/min for 1 h at 180 °C.

### 2.3. Attenuated Total Reflection Infrared Spectroscopy Experiment (ATR-FTIR)

**Figure 6:**
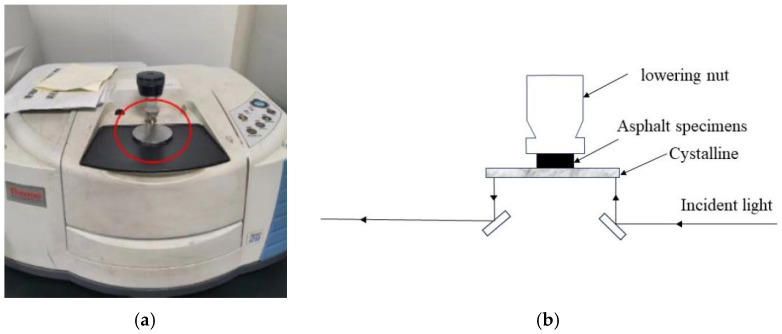
Process and principle of the ATR-FTIR experiments: (**a**) the process of testing, (**b**) principles of testing.

In order to test whether there is any chemical interaction in the interaction between fiber and asphalt in the process of two kinds of fiber-modified asphalt, as well as the difference in the ability of two kinds of fibers to adsorb asphalt, this paper tested two kinds of fiber-modified asphalt by using ATR-FTIR experiments. Furthermore, this article does not address the examination of optimum fiber doping, whereas Chen K et al. [29] investigated the optimum doping of two fibers. As a result, in this work, the ideal fiber dose inside the modified asphalt is determined throughout the testing phase. The test process and principle are shown in Figure 6:

### 2.4. Testing of Rheological Properties

The rheological qualities of viscoelastic materials refer to how the material’s viscoelasticity changes with temperature or load [30]. To analyze the difference in the rheological properties of the modified asphalt due to the difference in physicochemical interaction between the two fibers and the asphalt components, the rheological properties of the two fiber-modified asphalts were tested in this paper at the optimal blending amount, respectively.

#### 2.4.1. Testing of High-Temperature Rheological Properties

**Figure 7:**
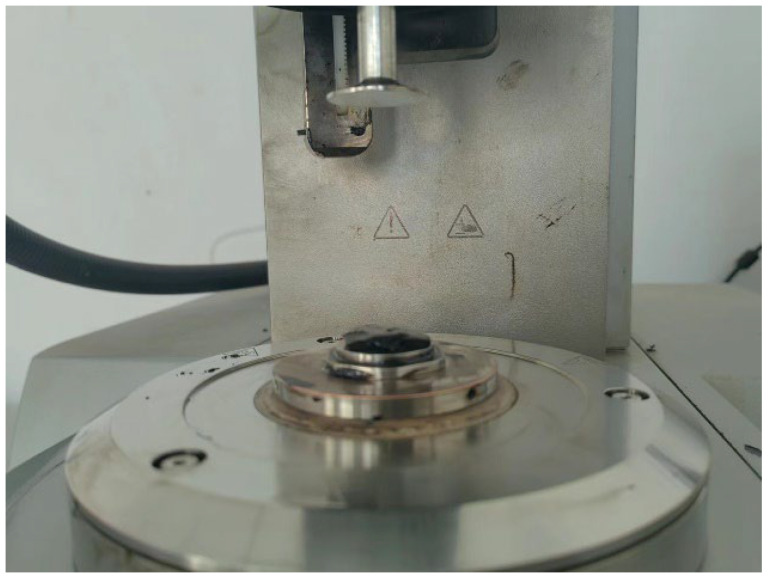
Equipment and specimens for temperature scanning tests.

The complex shear modulus (G*) and phase angle (°) are two metrics that describe how the viscoelasticity of asphalt changes as temperature increases [31]. The asphalt material’s high-temperature performance improves when the complex shear modulus increases and the phase angle decreases. This article investigated the high-temperature rheological characteristics of two fiber-modified asphalts using a dynamic shear rheometer. The test mode was temperature scanning, with a 12% control strain and a test temperature range of 46–82 °C in 6 °C increments. The test procedure is shown in Figure 7:

#### 2.4.2. Low-Temperature Performance Tests

In a low-temperature environment, the asphalt material produces internal temperature stress; if the stress relaxation capacity is insufficient, it will lead to cracking damage when subjected to load action. As a result, it is necessary to begin with the differences in the physicochemical modification mechanisms of two types of fibers on asphalt materials and to assess the impact of this difference on the low-temperature performance of asphalt materials.

In this research, the Bending Beam Rheometer test (BBR) was used to assess the creep modulus of the strength of the two fiber-modified asphalts at the optimal dose [32] to further examine the difference in low-temperature cracking resistance. The test temperatures were −6 °C, −12 °C, and −18 °C. The formula for the indicator is shown in Equation (4):

(4) St=Pl34bh3vt

where: P is the loads applied during the experiment (N); l, b, h is the length, width, and height of the specimen, respectively (mm); and v is the deformation value at the center of the test piece (mm)

**Figure 8:**
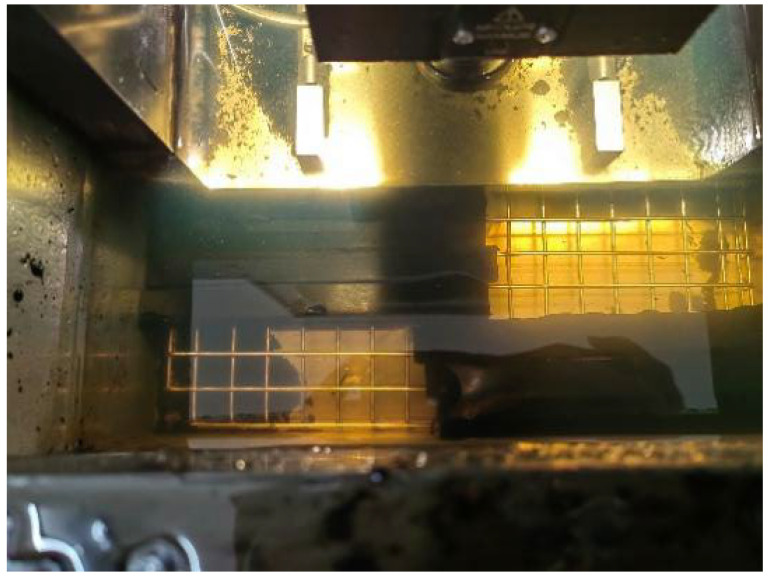
Test apparatus and process of low-temperature creep performance.

The test equipment and specimens are shown in Figure 8:

## 3. Results and Discussions

### 3.1. Analysis of Simulation Results of Molecular Physical Interaction Between Fibers and Bitumen

#### 3.1.1. Analysis of the Results of Model Runs

**Figure 9:**
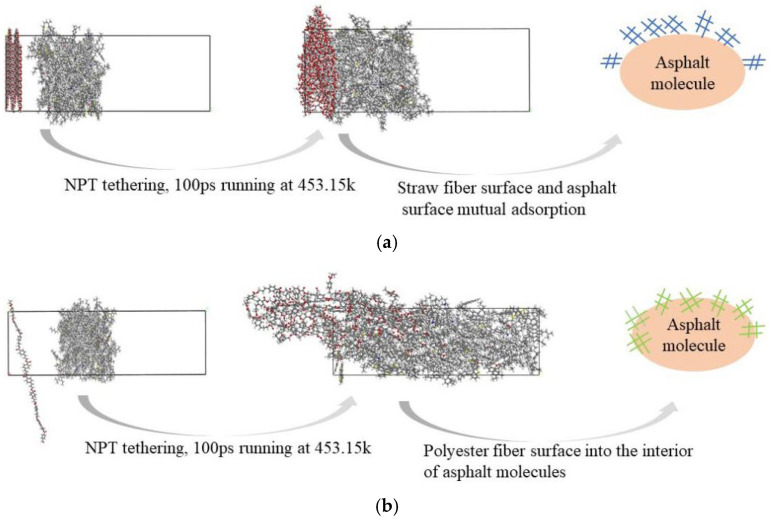
Straw/polyester fiber asphalt structural model run results and schematics. (**a**) Results and schematic of the run of the molecular structure model of straw fiber asphalt. (**b**) Results of the run of the polyester fiber asphalt molecular structure model and schematic diagrams.

Figure 9 depicts the final states of the straw fiber asphalt molecular structure system and the polyester fiber asphalt molecular structure system after 100 ps of operating time.

The results of the model runs and the schematic in Figure 9 show that after running for the same amount of time in the same environment, the surface of the straw fiber molecules adsorbed on the surface of the asphalt molecules and did not enter the interior of the asphalt molecules, whereas the chain structure of the polyester fiber molecules gradually entered the interior of the asphalt molecules. As a result, it is clear that the adsorption of polyester fiber molecules to asphalt, as well as penetration into the interior of asphalt molecules, is stronger than that of straw fibers. The phenomenon of polyester fiber molecules’ chain-like structure progressively entering the inside of asphalt molecules causes the polyester fiber molecules to have a greater surface area to adsorb the asphalt components, resulting in stronger interfacial sorption.

#### 3.1.2. Analysis of Differences in Molecular Physical Interaction Between Fiber and Asphalt Components

**Figure 10:**
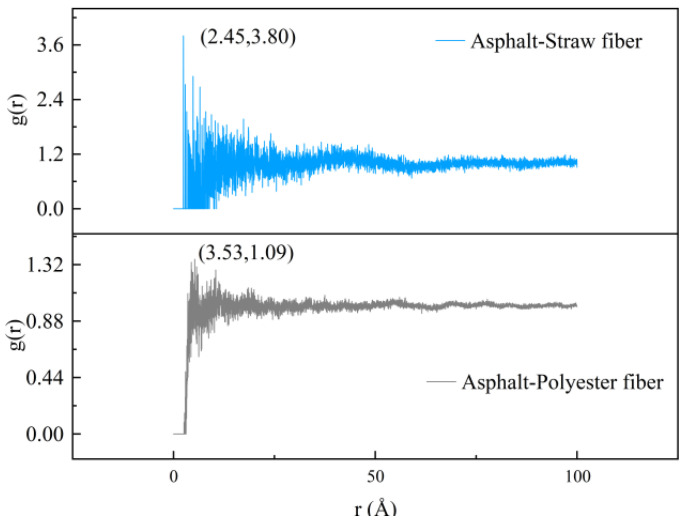
Radial distribution function between different fibers and bitumen.

Radial distribution functions (RDF) can be used in molecular dynamics to describe physical interactions between molecules (such as van der Waals interactions). In this study, the RDF between two types of fibers and asphalt was estimated individually using Materials Studio software (8.0), and the results are given in Figure 10.

The findings in Figure 10 show that the initial peak of the radial distribution function between straw fibers and asphalt occurs at 2.45 Å, whereas that between polyester fibers and asphalt occurs at 3.53 Å. Related studies have shown that the type of interaction between two molecules can be determined by the region in which the peak of the radial distribution function appears. When the peak region of the radial distribution function is less than 2.5 Å, the two molecules are hydrogen-bonded, and when it is greater than 2.5 Å, the interaction is van der Waals. As a result, it is possible to conclude that the straw fibers and the asphalt molecules form hydrogen bonds, whereas the polyester fibers and the asphalt molecules form van der Waals interactions. In this article, it is postulated that the variation in physical interactions between the molecules is caused by the various reactive groups on the surface of the straw fiber and polyester fiber. The presence of reactive groups on straw fibers’ surfaces, such as hydroxyl groups, permits hydrogen bonds to form with asphalt. Since the polyester fiber molecules include ester groups and aromatic rings, the physical contact with the asphalt component is defined by van der Waals interaction.

Furthermore, upon comparing the peak values of the radial distribution functions (RDFs) between straw fiber molecules and polyester fiber molecules with asphalt, it is observed that the RDF peak between straw fiber molecules and asphalt exhibits a higher value of 3.80, in contrast to the relatively lower peak of 1.09 observed for the RDF between polyester fiber molecules and asphalt. This finding underscores significant differences in the interaction characteristics between these fiber types and asphalt. The magnitude of the peak in the radial distribution function serves as an indicator of the intensity of intermolecular interactions; specifically, a larger peak signifies a stronger intermolecular interaction, whereas a smaller peak implies a weaker one. Therefore, it is plausible to deduce that the interaction between straw fiber molecules and asphalt surpasses that of polyester fiber molecules, which is attributed to the robust intermolecular interactions occurring between the hydroxyl groups present on the surface of straw fiber molecules and the asphalt, which form hydrogen bonds. However, the interaction between straw fiber molecules and asphalt is confined to the interfacial region only. In contrast, as evident from the model results depicted in Figure 9, polyester fiber molecules possess the capability to penetrate within the asphalt matrix, thereby forming a mechanically interlocked structure. The potential enhancement of modified asphalt performance as a result of this mechanically interlocked structure will be thoroughly analyzed through subsequent simulations and experimental procedures presented within this paper.

#### 3.1.3. Analysis of Differences in Spatial Distribution of Asphalt Fractions on Fiber Surfaces

**Figure 11:**
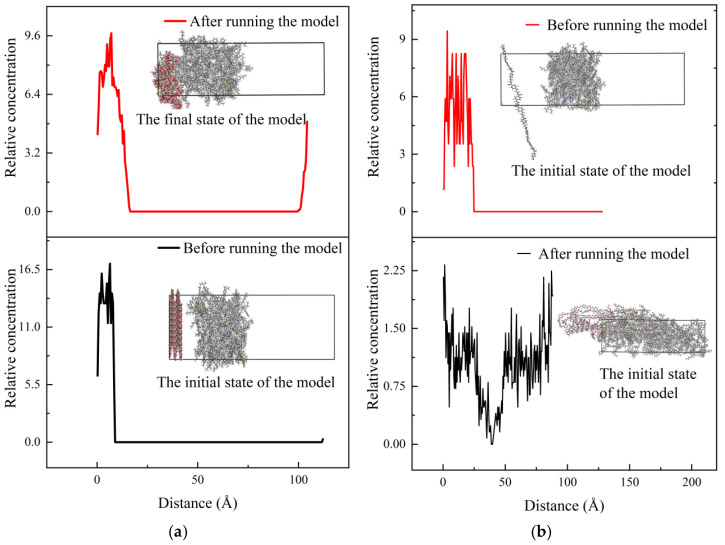
Relative concentration distribution of different fibers along the *z*-axis: (**a**) asphalt–straw fiber, (**b**) asphalt–polyester fibers.

The results of the model runs in Figure 9 reveal that the two types of fibers have distinct diffusion capabilities at the surface and inside the asphalt molecules. To further analyze the changes in diffusion capacity caused by the differences in the interactions between the two fibers and the asphalt molecules, this paper calculates the concentration distributions of polyester and straw fibers along the asphalt (*z*-axis direction) before and after the model runs, respectively. Specifically, hydroxyl groups (-OH) were selected to represent straw fiber molecules, while carboxyl groups (-COOH) were chosen to represent polyester fiber molecules. The results of the calculations are shown in Figure 11:

The concentration distribution curve along the *z*-axis in Figure 11 demonstrates that before and after the model run, the concentration of polyester fibers along the *z*-axis varies more dramatically. Polyester fiber molecules vary from 0 to 100 Å. Straw fibers were distributed in the range of 0–24 Å. This indicates that polyester fibers are more likely to diffuse into the core of asphalt molecules, whereas straw fibers can only absorb asphalt on their surfaces and have a lower diffusion capability. This article hypothesizes that it is due to the variations in the molecular structure of straw fibers and polyester fibers. Both polyester fibers and bitumen include aromatic ring structures, which can attract one other through β-electron production. Furthermore, the aromatic rings are stable and have stable intermolecular interactions with the bitumen, allowing the polyester fibers to permeate into the bitumen molecules. The adsorption of asphalt by straw fiber is solely dependent on its surface hydroxyl and other active groups, as well as the creation of hydrogen bonds between the asphalt and other physical effects.

#### 3.1.4. Analysis of the Effect of Differences in Molecular Physical Interaction on the Diffusion Properties of Asphalt Components

**Figure 12:**
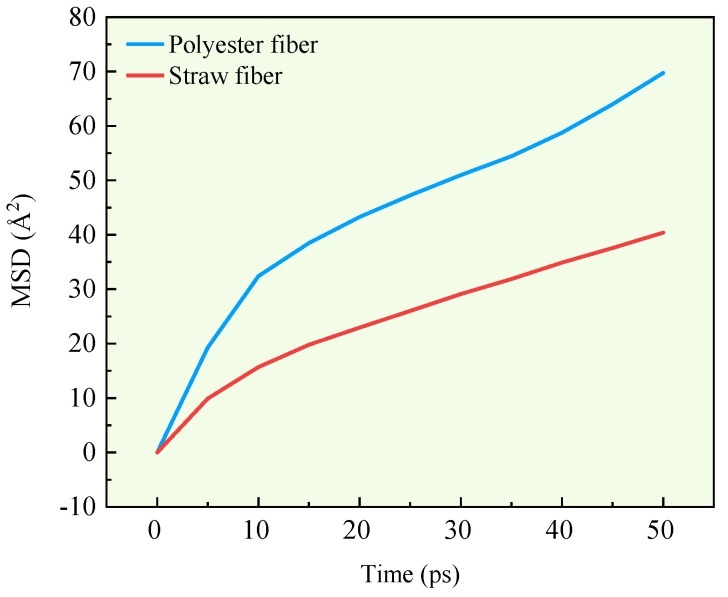
MSD curves of two fibers in asphalt-fiber system.

In molecular dynamics, the capacity of a molecule to diffuse in a system may be assessed by a mean square displacement curve and diffusion coefficient. In this research, the mean square displacement curves of the fibers after running the two fiber asphalt structural models are computed, and the results are given in Figure 12.

Following computation, the slopes of the mean square displacement curves for polyester and straw fibers are 0.891 and 0.6, respectively. Thus, the diffusion coefficients of the two fibers are 0.1485 and 0.1. Based on the findings of the mean square displacement curve and diffusion coefficient, polyester fiber has a 32.67% higher diffusion coefficient than straw fiber. Thus, research shows that polyester fibers have a higher diffusion capability than straw fibers in the fiber asphalt structural system. The higher the diffusion capacity, the greater the contact between the fibers and the asphalt, and the more surface area available to adsorb the asphalt molecules.

### 3.2. Analysis of Differences in Chemical Interaction Between Two Fiber Surfaces and Bitumen

**Figure 13:**
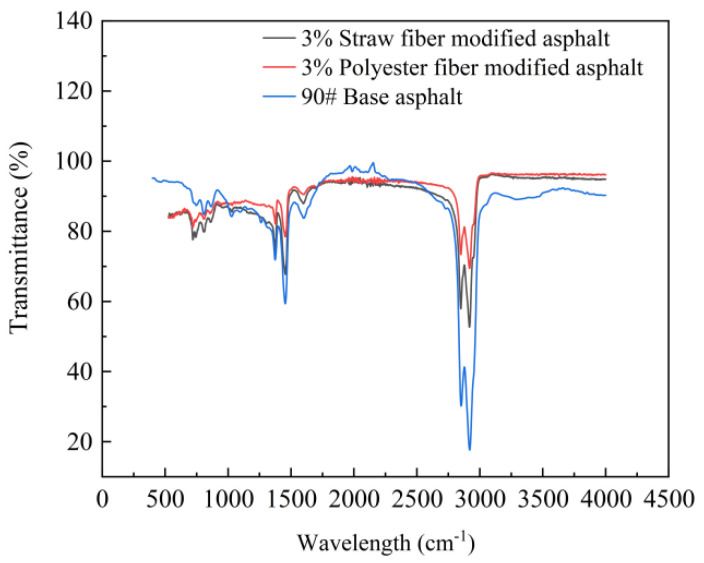
Infrared spectral test results of different bitumen.

This paper used Fourier Transform Infrared Spectroscopy (FTIR) technology to test the two types of fiber-modified asphalt and 90# matrix asphalt in order to compare and analyze the chemical differences between the two types of fibers and asphalt, as well as the chemical changes that occur during the process of modifying asphalt to create new functional groups. The test results are shown in Figure 13:

Figure 13 shows that the distinctive peaks in the infrared spectra of the two fiber-modified asphalts are identical to those of the 90# base asphalt. This means that the two fibers are simply physically mixed in the asphalt and that there is no chemical interaction between the fibers and the asphalt. Furthermore, the infrared light transmittance of the functional groups varied among the two fiber-modified asphalts and the matrix asphalt. This article hypothesizes that this is connected to the adsorption of asphalt components by fibers. Fiber adsorption of asphalt components, fiber-modified asphalt specimens on the surface of the concentration of different asphalt components, and the spatial distribution of asphalt components along the surface of the fiber to the surface of the fiber-modified asphalt in the region of the asphalt components will differ. The findings of the infrared spectroscopy tests show variances in the concentration of the bitumen’s distinctive functional groups.

In addition, when the infrared spectra of straw fiber-modified asphalt and polyester fiber-modified asphalt at the optimal dosage are compared, the infrared transmittance of polyester fiber-modified asphalt is greater than that of straw fiber-modified asphalt, implying that the concentration of functional groups in the surface area of polyester fiber-modified asphalt under test is lower than that of straw fiber-modified asphalt. This research speculates that the cause of this phenomenon is the molecular structure of the two fibers. Straw fiber is mostly composed of polymerized glucose monomer molecules, with reactive groups such as hydroxyl, whereas polyester fiber consists primarily of ester groups and aromatic rings. The presence of ester groups causes the chain structure of polyester fiber molecules to entangle and lap each other, resulting in a stable configuration for bitumen adsorption [33]. Both asphalt and polyester fibers include aromatic rings, which can form stable contacts (e.g., van der Waals, Π–Π stacking) [34]. This facilitates the asphalt component’s infiltration and diffusion over the surface of the polyester fiber chain structure, eventually encapsulating the polyester fiber to produce a stable interface. Although the monomer structure of straw fiber has more reactive groups, such as hydroxyl groups, the components that play an elastic function in asphalt (for example, asphaltenes and gums) are nonpolar molecules. The electron cloud on its surface is more evenly distributed, making it more difficult to make hydrogen bonds with the straw strand. As a result, straw fibers have a lower adsorption capacity for asphalt components than polyester fibers.

In conclusion, while there is no chemical interaction between the two types of fibers and asphalt, the adsorption capacity of the asphalt components varies due to changes in their chemical molecular structures, affecting the performance of the modified asphalt.

### 3.3. Analysis of the Effect of Differences in Physicochemical Interactions on the High-Temperature Performance of Modified Asphalt

#### 3.3.1. Differential Analysis of the Impact of Physicochemical Interaction Variations on the Complex Shear Modulus of Modified Asphalt

**Figure 14:**
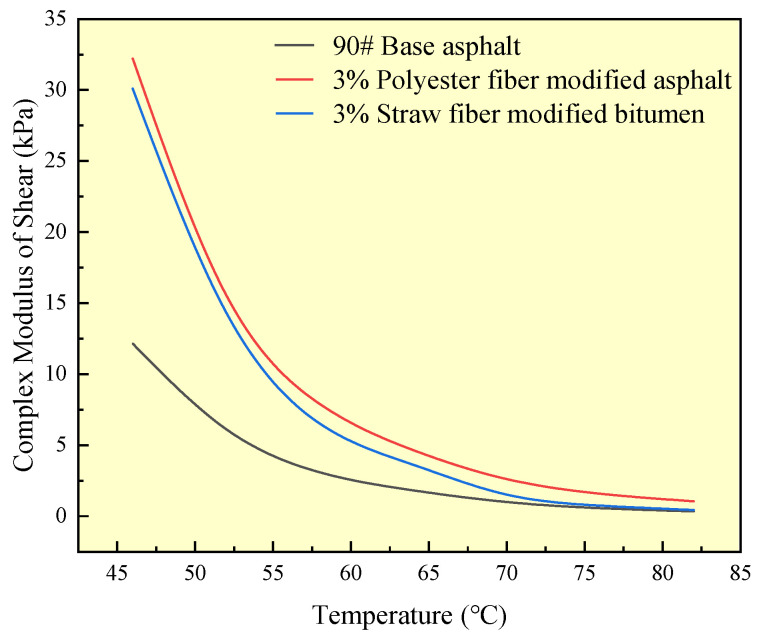
Complex shear modulus test results for different asphalts.

The complex shear modulus (G*) may indicate the high-temperature deformation resistance of asphalt materials; the higher the value, the stronger the deformation resistance of asphalt materials; when asphalt materials are in high-temperature conditions, the viscosity is more difficult to diminish. A dynamic shear rheometer was used in this study to measure the complex shear modulus of two kinds of fiber-modified asphalts and matrix asphalt. The test results are shown in Figure 14:

As shown in Figure 14, the complex shear modulus of all three asphalts drops as temperature increases, and the slope of the curve steadily diminishes. This indicates a progressive shift from all three asphalt states to flow dynamics. In the temperature range of 46 °C to 82 °C, the complex shear modulus of both fiber-modified asphalts exceeded that of 90# base asphalt. This was due to the adsorption of polyester fibers and straw fibers in the modified asphalt on the asphalt components, limiting the asphalt components’ capacity to flow as temperature increased.

When the complex shear modulus of polyester fiber-modified asphalt and straw fiber-modified asphalt are compared, it is clear that polyester fiber-modified asphalt has a higher complex shear modulus than straw fiber-modified asphalt over the whole temperature range of the test. This paper hypothesizes that this is connected to the difference in the physicochemical mechanism of action of the two fibers in their impact on asphalt components. Straw fiber adsorption of asphalt components is primarily caused by the physical contact between the fiber and the asphalt components, such as van der Waals interactions. In addition to the physical interaction between polyester fibers and asphalt components, the active groups on the surface of the fibers (e.g., carboxyl groups, etc.) and the components of the asphalt and chemical adsorption (e.g., the carboxyl groups on the surface of the polyester fibers and the carboxyl groups in the components of the asphalt through hydrogen bonding to form a carboxyl dimer) have an impact. This will result in a stronger interfacial connection between the polyester fiber and the asphalt component than the straw fiber. At the same time, the polyester fiber and asphalt components will not interact, and the chemical reaction is not harmful to the asphalt, indicating high chemical compatibility. Stronger interfacial bonding and chemical compatibility improve the polyester fiber adsorption of asphalt components, resulting in superior high-temperature performance of the polyester fiber-modified asphalt than straw fiber-modified asphalt.

#### 3.3.2. Analysis of the Effect of Physicochemical Interaction Differences on the Phase Angle of Modified Bitumen

**Figure 15:**
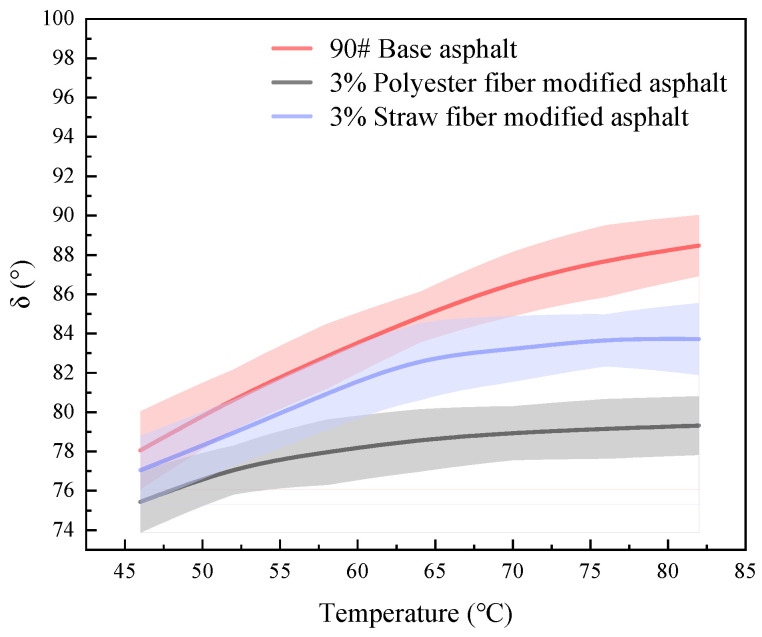
Phase angle test results of different modified asphalt.

The phase angle (°) may also be used to determine the high-temperature qualities of asphalt materials. The phase angle relates to the ratio of viscoelastic components in the asphalt; the bigger the phase angle, the greater the proportion of viscous components in the asphalt and the fewer elastic components; at the same time, the greater the flow capacity of asphalt, the poorer the resistance to deformation. In this article, the phase angles of 90# matrix asphalt, straw fiber-modified asphalt, and polyester fiber-modified asphalt were investigated in the temperature range of 46 °C to 82 °C using a dynamic shear rheometer. Three samples of each asphalt were evaluated, and the findings were averaged. The test results are shown in Figure 15:

As can be seen from the data in Figure 15, the magnitude of the phase angles of the three modified bitumens in the temperature range of 46–82 °C is in the following order: 90# base asphalt > 3% straw fiber-modified asphalt > 3% polyester fiber-modified asphalt. In practice, 64 °C is closer to the real temperature of the road surface. When comparing the phase angles of the two fiber-modified asphalts at 64 °C, the polyester fiber-modified asphalt had a 4.289° lower phase angle than the straw fiber-modified asphalt. This suggests that polyester fiber-treated asphalt has more elastic components than straw fiber-modified asphalt at this specific temperature.

Furthermore, when comparing the degree of change in phase angle of the three types of asphalt, the average change in phase angle for 90# matrix asphalt, polyester fiber-modified asphalt, and straw fiber-modified asphalt was 10.41°, 3.88°, and 6.672°, respectively. According to this, the rate of loss of elastic ingredients in 90# matrix asphalt is the biggest, followed by straw fibers, and polyester fibers are last as the temperature rises in the same temperature range.

This research hypothesizes that this is due to the fibers’ adsorption of asphalt components and the variations in the two fibers’ physicochemical adsorption action principles. The presence of fibers adsorbs asphalt components, reducing the pace of temperature-induced viscoelasticity shift. At the same time, due to the difference in physicochemical effects between the two fiber surfaces and asphalt, there are certain reactive groups (e.g., hydroxyl) on the surface of straw fibers, which might have generated hydrogen bonding effects with the asphalt component. The active groups on the surface of polyester fibers are ester groups. Thus, the contact between the reactive groups in the fibers and the bitumen should have been greater. However, the phase angle changes for polyester fiber-treated asphalt are substantially lower than those for straw fiber-modified asphalt. The study speculates that this is due to the involvement of ester groups in polyester fibers and the difference in chemical polarity between polyester and straw fibers.

Straw fibers are extremely polar due to the presence of hydroxyl and other reactive groups, whereas polyester fibers with ester groups and aromatic rings are non-polar, as is asphalt. Because the electron cloud on the asphalt component’s surface is more consistently dispersed, the hydroxyl group of straw fiber takes more energy to breach the electron cloud barrier while forming a hydrogen bond with the asphalt component. As a result, despite the presence of active groups such as hydroxyl groups on the surface of straw fiber, the dipole moment on its surface cannot create significant interactions with asphalt components. In contrast, van der Waals interactions can be created between the nonpolar groups in the polyester fiber and the nonpolar groups in the asphalt. Although the strength of van der Waals interactions is inherently lower than that of hydrogen bonding, polyester fibers and asphalt can form a stable and temporary dipole moment, and the dipole moments can be attracted to each other, allowing polyester fibers to adsorb asphalt components more effectively than straw fibers.

As a result, with increasing temperature, the diffusion ability of polyester fiber-modified asphalt components is weaker than that of straw fiber-modified asphalt, the rate of reduction of elastic component content is slower, the phase angle is larger and the rate of change is slower, and high-temperature performance is superior.

### 3.4. Analysis of the Effect of Physicochemical Interaction Differences on the Low-Temperature Performance of Modified Asphalt

Because of the various groups in polyester and straw fibers, there will definitely be variances in the molecular structure of the fibers as well as qualities such as hardness, resulting in discrepancies in the low-temperature performance of fiber-modified asphalt. To investigate the effects of differences in physicochemical interactions between polyester and straw fibers and asphalt components on the low-temperature properties of asphalt materials, this paper used BBR experiments to measure the low-temperature creep capacity of the two types of fiber-modified asphalt at the optimal blending level, which was quantitatively represented by the creep modulus of strength.

**Figure 16:**
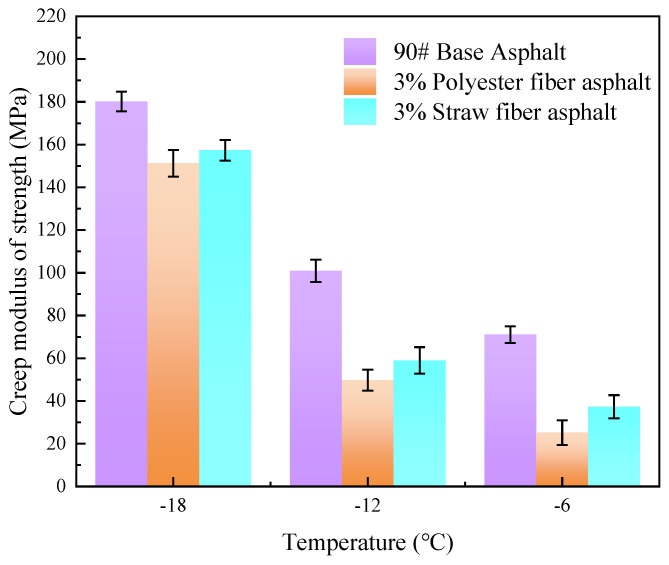
Creep strength of different fiber-modified asphalt.

The creep modulus of strength refers to the capacity of an asphalt material to withstand deformation when applied to a load. In other words, the stiffness of the asphalt. The higher the creep modulus of strength, the stiffer the asphalt and the more susceptible it is to brittle fracture in low-temperature conditions. In this research, the creep strength moduli of 90# base asphalt, 3% blended polyester fiber-modified asphalt, and 3% blended straw fiber-modified asphalt were examined at three temperatures, −6 °C, −12 °C, and −18 °C, using BBR experiments. The test results are presented in Figure 16.

Figure 16 shows that the creep strength modulus of the three asphalts at three temperatures is 90# basic asphalt > 3% straw fiber-modified asphalt > 3% polyester fiber-modified asphalt. At three temperatures (−6 °C, −12 °C, and −18 °C), the creep strength modulus of 3% straw fiber-modified asphalt and 3% polyester fiber-modified asphalt decreased by 47.5%, 41.52%, 12.67%, and 64.55%, 50.71%, and 16.05%, respectively, compared to 90# base asphalt. It is clear that adding fibers into asphalt may significantly lower the low-temperature creep modulus of asphalt materials. This is because, when fibers are added to asphalt in a low-temperature environment and subjected to the action of a load, the fibers gain a certain degree of strength, which can aid in load transmission and increase the low-temperature cracking resistance of asphalt materials.

When comparing the creep modulus of polyester fiber-modified asphalt and straw fiber-modified asphalt, the creep modulus of polyester fiber-modified asphalt is smaller than that of straw fiber-modified asphalt by 32.48%, 15.72%, and 6.09%, respectively, under the three low-temperature conditions (−6 °C, −12 °C, and −18 °C). Although both polyester fiber and straw fiber are fibrous materials, polyester fiber-modified asphalt has higher low-temperature cracking resistance than straw fiber-modified asphalt. Again, this is due to the physicochemical action of the two adsorbing bitumens and the chemical structure of the fiber. The difference in the physicochemical effects of the two adsorptions of asphalt compared with straw fiber, polyester fiber in the aromatic ring structure, and asphalt components may establish a stable adsorption interface. Because the molecular polarities of straw fiber monomers and asphalt molecules disagree, stable interfacial adsorption between straw fiber and asphalt molecules is difficult to achieve. When the two fiber-modified asphalts are loaded at low temperatures, the stress transmission between them differs. Polyester fiber and asphalt interface adhesion leads to more excellent stress transmission, so because of the polyester fiber-modified asphalt in the load, the stress may be completely transmitted from asphalt to polyester fiber. Because of the low interfacial adhesion in straw fiber-modified asphalt, stress cannot be efficiently transmitted to the straw fibers when loaded, resulting in high stresses inside the asphalt. Furthermore, polyester fiber molecules have a significant number of ester groups, giving the polyester fiber molecular chain structure a high degree of flexibility. This enhances the capacity of neighboring polyester fiber molecules to interweave with one another to produce a stable mesh structure inside the asphalt. As a result, polyester fibers can tolerate more strains than straw fiber molecules.

In conclusion, because of the difference in the chemical structure of polyester fiber and straw fiber molecules, as well as the difference in the adsorption capacity of asphalt caused by the difference in the structure of molecules, polyester fiber-modified asphalt performs better at low temperatures than straw fiber-modified asphalt.

## 4. Conclusions

In this paper, the differences in physicochemical interactions between polyester and straw fibers and asphalt, as well as the effects on the properties of the two fiber-modified asphalts, were thoroughly analyzed using molecular simulation techniques, infrared spectroscopy, and rheological property tests, and the following conclusions were attained:

1. According to the results of molecular simulation, there is van der Waals interaction between polyester fiber and asphalt, and there is primarily hydrogen bonding between straw fiber and asphalt; however, the diffusion ability of polyester fiber in asphalt is greater than that of straw fiber, allowing the polyester fiber molecular chain structure to adsorb asphalt components over a larger area.
2. Based on the infrared spectroscopic findings of 90# matrix asphalt, polyester fiber asphalt, and straw fiber-modified asphalt, there is no chemical interaction between the two fibers and the asphalt components or adsorption of asphalt components for physical adsorption. However, due to the aromatic ring structure contained in the chain structure of the polyester fiber molecule, as well as the molecule’s non-polar properties, its ability to adsorb the asphalt component is much stronger, resulting in the highest transmittance in the infrared spectroscopy tests.
3. Temperature scanning experiments on 90# base asphalt, polyester fiber-modified asphalt, and straw fiber-modified asphalt revealed that polyester fiber-modified asphalt has the highest complex shear modulus, followed by straw fiber-modified asphalt, and 90# base asphalt has the lowest complex shear modulus. The order of magnitude of the phase angle is as follows: 90# matrix asphalt, straw fiber-modified asphalt, and polyester fiber-modified asphalt. The polyester fiber-modified asphalt has a lower phase angle than the straw fiber-modified asphalt by 4.289° at 64 °C. This suggests that the polyester fiber molecules’ excellent chemical structure, as well as their differences in functional groups from straw fiber molecules, allow the polyester fibers to adsorb more asphalt fractions and reduce the diffusion rate of the asphalt fractions at elevated temperatures, thereby improving high-temperature performance.
4. Based on the low-temperature performance test results of 90# base asphalt, polyester fiber-modified asphalt, and straw fiber-modified asphalt, the advantages and disadvantages are in the order of: polyester fiber-modified asphalt > straw fiber-modified asphalt > 90# base asphalt. The stress transfer capacity of the interface generated by adsorption between polyester fibers and asphalt is greater than that of the adsorption contact between straw fibers and asphalt. The creep modulus of strength of polyester fiber-modified asphalt was 32.48%, 15.72%, and 6.09% lower than that of straw fiber-modified asphalt at three low temperatures of −6 °C, −12 °C, and −18 °C, respectively.

In summary, because polyester fiber and straw fiber have different chemical structures and active groups, polyester fiber-modified asphalt performs better than straw fiber-modified asphalt, and the aromatic ring and ester group structures in polyester fiber play an important role in the modification process.

## Figures and Tables

**Table 1:** Technical index test results of matrix asphalt.

Technical Indicators	Unit	Technical Requirement	Results of the Test	Methodology of the Test
Penetration (25 °C, 5 s, 100 g)	0.1 mm	80–100	89	T0604
Softening point (T&B)	°C	≥43	45.2	T0606
Ductility (10 °C)	cm	≥45	49.3	T0605

**Table 2:** Technical specifications of rice straw fiber.

Length (mm)	Density (g/cm^3^)	Caliber (μm)	Tensile strength (MPa)	Bending Modulus of Elasticity (MPa)	pH-Value
4–6	1.33	40–450	44.5	4678.2	7.2

**Table 3:** Technical specifications of polyester fibers.

Length (mm)	Density (g/cm^3^)	Caliber (μm)	Tensile Strength (MPa)	Elongation at Break (%)	Modulus of Elasticity (MPa)	Melting Point (°C)	Combustion Point (°C)
6	1.38	25	≥600	≥15	≥8000	258	556

## Data Availability

The original contributions presented in the study are included in the article, further inquiries can be directed to the corresponding author.
